# From rationality to cooperativeness: The totally mixed Nash equilibrium in Markov strategies in the iterated Prisoner’s Dilemma

**DOI:** 10.1371/journal.pone.0180754

**Published:** 2017-11-30

**Authors:** Ivan S. Menshikov, Alexsandr V. Shklover, Tatiana S. Babkina, Mikhail G. Myagkov

**Affiliations:** 1 Department of Control and Applied Mathematics, Moscow Institute of Physics and Technology (State University), Moscow, Moscow Region, Russian Federation; 2 Department of Mathematical Modeling of Economic Systems, Dorodnicyn Computing Center, Federal Research Center «Computer Science and Control» of Russian Academy of Science, Moscow, Moscow Region, Russian Federation; 3 Center for Design, Manufacturing and Materials, Skolkovo Institute of Science and Technology, Moscow, Moscow Region, Russian Federation; 4 Laboratory of Experimental Methods in Cognitive and Social Sciences, Tomsk State University, Tomsk, Tomsk Region, Russian Federation; 5 Department of Political Science, University of Oregon, Eugene, Oregon, United States of America; Groupe ESC Dijon Bourgogne, FRANCE

## Abstract

In this research, the social behavior of the participants in a Prisoner's Dilemma laboratory game is explained on the basis of the quantal response equilibrium concept and the representation of the game in Markov strategies. In previous research, we demonstrated that social interaction during the experiment has a positive influence on cooperation, trust, and gratefulness. This research shows that the quantal response equilibrium concept agrees only with the results of experiments on cooperation in Prisoner’s Dilemma prior to social interaction. However, quantal response equilibrium does not explain of participants’ behavior after social interaction. As an alternative theoretical approach, an examination was conducted of iterated Prisoner's Dilemma game in Markov strategies. We built a totally mixed Nash equilibrium in this game; the equilibrium agrees with the results of the experiments both before and after social interaction.

## Introduction

The traditional approach to analyzing the decision-making process of the participants in game-like interaction is based on the individual rationality principle of each participant [1,2]. The Nash equilibrium and its numerous generalizations postulate the principle of the best response by each participant in the interaction to the behavior of others [3–5].

Such an approach enabled creating and researching numerous models of social and economic behavior noted, in particular, by several Nobel prizewinners in economics. At the same time, extensive empirical and experimental data on game-like interaction have been accumulated. In these data people’s behavior cannot be explained only from the individual rationality position [6–8]. Thus, we must consider the social characteristics of decisions taken:

1. cooperation as contrary to individualism [9,10];
2. fairness, based on non-acceptance of inequality [9,11,12];
3. trust and gratefulness [13,14]; and
4. level of social responsibility [15,16].

One of the standard methods in the theoretical description of data that does not correspond to the theory of rationality is a quantal response equilibrium (QRE) model. To date there have been several attempts at using the QRE approach in the analysis of experimental data. In [17] it was found that the experimental data on auctions is well interpreted using QRE. Moreover, in [18] it was shown that a QRE model complements the method of maximum likelihood by considering the irrationality of the players participating in experiments. The application of QRE to 2×2 games was researched in [4]. Another approach is the method of Markov chain introduction, demonstrated in [19]. The main problem in such research is the originality of the behavioral experimental data in each piece of research. This demands an individual approach and theoretical basis. In this paper we explain how a QRE model and Markov chains were applied to the data of real experiments with the Prisoner’s Dilemma game and the Trust Game.

## Materials and methods

### Participants

To analyze the social characteristics of people’s behavior during game-like interaction in small groups (4–12 subjects), numerous experiments were conducted in 2013–2016 at the Laboratory of Experimental Economics (LEE) at the Moscow Institute of Physics and Technology (MIPT) in cooperation with the Skolkovo Institute of Science and Technology in 2013–2016, that clearly reveal one or another social characteristic of behavior. In this paper, the results of eight experiments are presented. In each of them, the number of participants was equal and consisted of 12 people; thus, the data on 96 participants (59 males, 37 females) were taken into consideration. For each experiment, MIPT students who were unknown to each other were selected as participants. Characteristics such as major, group, and year of studies were considered during the selection. Recruitment was by advertisements in the VKontakte social network (vk.com). Skolkovo Institute of Science and Technology Human Subjects Committee approved the study procedures involving human participants. Written informed consents were obtained from participants. Experimental data are readily available on Harvard Dataverse: http://dx.doi.org/10.7910/DVN/ZGW6ZP.

### Design and procedures

**Fig 1:**
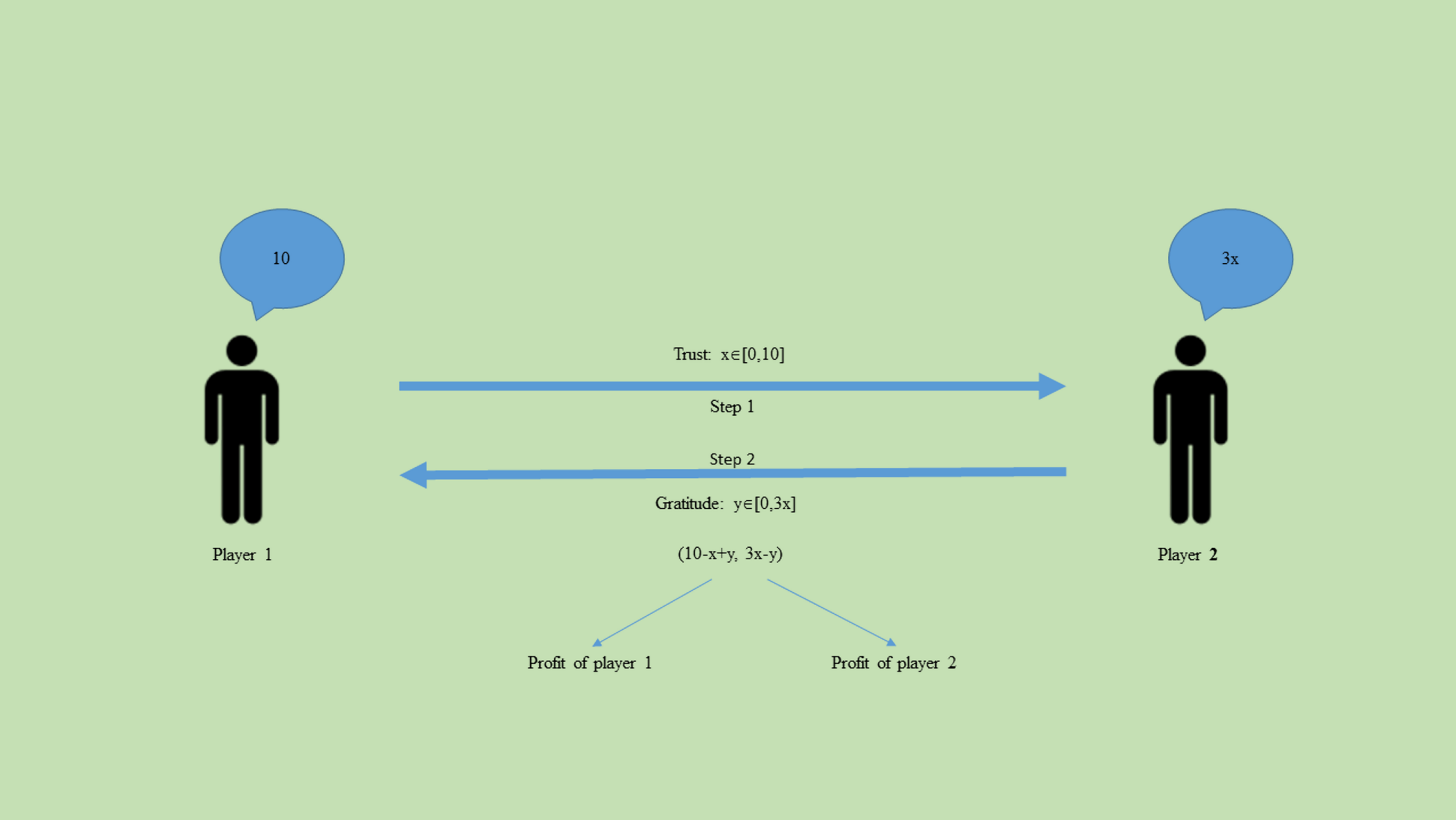
The structure of the Trust Game. The illustration represents the decision making process during the TG. Player 1 starts the game and offers some integer number from zero to ten to player 2. Player 2 gets the offered number multiplied by three. Then player 2 returns any number of the number available.

During the experiment the participants were asked to play the following:

1. Prisoner’s Dilemma (PD). Each of two participants has two strategies: Cooperation (Up or Left) or Defection (Down or Right). In the standard PD, two players are offered the same points, R, for Cooperation and a smaller gain, P, for Defection. If one of the players cooperates and another defects, the cooperator gains a smaller reward, T, but the defector takes a larger reward, S. Thus, there is a ration between prizes T>R>P>S (Table 1) [6]. Defection is more profitable than Cooperation in any partner’s choice, but mutual Cooperation is more profitable for both than mutual Defection. The Nash equilibrium corresponds to mutual Defection (P, P), but the participants try to establish mutual Cooperation (R, R) [20].
2. Trust and Gratefulness (Trust Game) (TG). One of the participants (the Grantor) can entrust another participant (the Grateful) with some of his or her own money (from 0 to 10). The money obtained (invested) is tripled and the Grateful can share any part of this increased amount with the Grantor (Fig 1). In the totally mixed Nash equilibrium, there is no sense in gratefulness, and therefore there is no sense in trust, which leads to a zero result for both participants [21,22].

**Table 1:** Prisoner’s Dilemma payoffs.

Payoffs	*Cooperation*	*Defection*
*Cooperation*	**R, R**	**S, T**
*Defection*	**T, S**	**P, P**

For the experiments considered in this paper, the parameters of the PD were set as R = 5, P = 1, S = 0, T = 10 (10 > 5 > 1 > 0).

In the experiments, gratefulness and trust on average are significantly greater than zero.

Each experiment was divided into three parts:

Part 1, the Anonymous stage. The participants were invited to play 11 rounds in PD at first and then 11 rounds in TG. A specialized tool to design and carry out group experiments in experimental economics, z-Tree developed at the University of Zurich, was used [23]. The participants were able to move to the next round only after all 12 participants made their choices. No one knew who their opponents were and in each round the pairs of participants changed randomly. After each round, the result of the round and the overall result for the current point in the game were displayed on the monitor.

**Fig 2:**
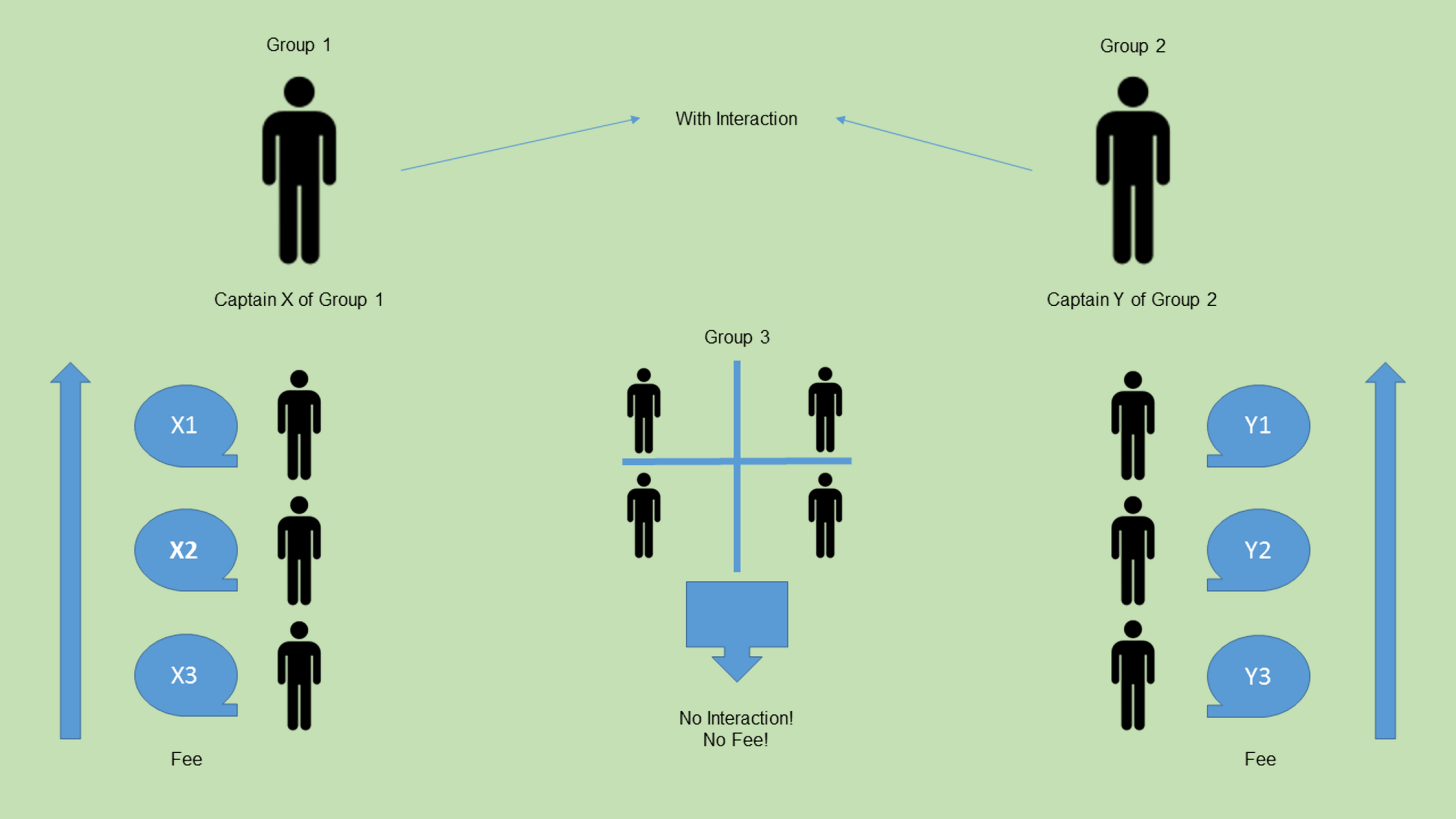
The illustration of the group formation during the Socialization stage. Two captains are chosen as volunteers from among the participants. Other participants must choose the captain whose team they want to join and how many points they are prepared to pay for that. The four participants, who paid less than the others, continue as individual participants in Group 3; they are forbidden to communicate or even look at each other. The participants in the teams with captains form Group 1 and Group 2.

Part 2, the Socialization stage. The participants were invited to take part in interactive cooperation. First the participants memorize each others’ names with the help of a snowball game: they sit in a circle, the first one gives his or her name and a personal characteristic that starts with the same letter as the name, the next participant repeats the name and the characteristic of the first participant and says his or her name and characteristic; then along the chain the game comes to the last person in the circle, who repeats all the names and all the characteristics. Then the participants in reverse order share their personal information: hometown, major, hobby, and interests. Then two captains are chosen as volunteers from among the participants. Other participants must choose the captain whose team they want to join and how many points they are prepared to pay for that. The participants find out their gain for the first part of the game. Then each of the participants except the captains must write on a piece of paper the name of the chosen captain and a number of points from, 0 to 50, that they are ready to pay in order to join the team of the chosen captain. The pieces of paper are personally given to the organizer, who sorts the piece by captains and points. In this way, two teams of four people with captains are formed. The remaining four participants, who paid less than the others, continue as individual participants; they are forbidden to communicate or even look at each other (Fig 2). The participants are informed about the procedure of distribution by teams beforehand, so all the steps are considered as circumspect and deliberate. At the end of the Socialization stage, the participants in the teams with captains have five minutes to find five common characteristics (eye color, favorite food, movie, etc.) and decide the name of the team.

Part 3, the Socialized stage. After Socialization, the participants are divided into three groups: two groups of four participants with captains and the four participants remaining. In this stage, the participants play PD and TG for 18 rounds within each group, i.e., the participants of group 1 with the captain played only with each other, it was the same for group 2, and the four participants remaining also played only with each other.

Therefore, we have the behavioral data of participants before Socialization in the general group of 12 people and after Socialization in the respective groups of four.

### Recent findings

In our study, we focus on the issues connected with the mechanism promoting cooperation. It was shown that the cooperation can be boosted by heterogeneous coupling between interdependent lattices [24], the link weight mechanism [25], and the size of the interaction neighborhood [26]. These findings explain the evolution of cooperation, especially the emergence of cooperation in the non-cooperative games such as PD [27].

Another approach to investigating cooperation is proposed in [1,28–30], where it was shown that incorporating of Socialization in the experiment in PD increases cooperation. The average level of cooperation in Part 1, the Anonymous stage, is 21%, whereas in Part 3, the Socialized stage, the average level of cooperation in the socialized groups is 53% [28]. From the viewpoint of the theory of rationality, as we know, the participants should not choose the strategy of cooperation; therefore the explanation of participants’ behavior in social experiments of this kind does not fit classic economic theory [31]. The increased level of cooperation is explained with the help of incorporating an additional component of the utility function—the social one. In this way, general utility consists of economic (rational) and social utility. The social component is understood as the completion of a socially useful accomplishment. For example, the cooperative move of a participant gives equal gain to an opponent that leads to the increase of social utility. However, it was of interest to us to elicit how the obtained data agrees with other well-known models, so we turned to the idea of QRE.

### About quantal response equilibrium

In this section we will discuss the attempts to explain the deviation of the results observed of participants’ behavior from the theoretical Nash equilibrium on the basis of the concept of QRE. We will note that the QRE conception appeared at the intersection of game theory and experimental economics in order to explain behavior of participants in the laboratory experiments that was significantly different from the Nash equilibrium [32,33].

“QRE is an internally consistent equilibrium model, in the sense that the quantal response functions are based on the equilibrium probability distribution of the opponents’ strategy choices rather than simply on arbitrary beliefs the players could have about those probabilities” [32]. One of the model’s features is that it allows game modeling of players who make mistakes. QRE imposes a requirement that beliefs should correspond to the equilibrium choice of probabilities. In this way, QRE demands solutions in the fixated point of choices of probabilities similar to the Nash equilibrium. However, unlike the classic Nash equilibrium, QRE supposes that the pursuit for the best response is realized by participants only in the probabilistic sense: the better the answer is, the higher the probability that it will be chosen by a participant [10,34].

“The QRE has been compared with experimental observation and generally provides a better fit to the data than the NE” [35]. On this basis, we decided to evaluate the model using our experimental data.

According to [33], we introduce QRE through the logistic quantal response function:

$$\sigma_{ij}\left(u_{i}^{*}\right)=\frac{e^{\lambda u_{ij}}}{\sum_{k=1}^{J_{i}}e^{\lambda u_{ik}}}$$ (1)

Here u_ij_ is the expected payoff of player i with strategy j, (j∈{1,…,J_i_}), $u_{i}^{*}=(u_{i1},\ldots ,u_{iJ_{i}})$ “If each player uses a logistic response function, QRE or logit equilibria are the solutions of Π_ij_ = α_ij_, where Π_ij_ is the frequency of strategy j in player i” [36].

### QRE in the PD game

For the PD game that we considered, the QRE (Table 1) could be determined as follows. Let p be the probability of the cooperative move of a partner, then the expected gain from the cooperative action equals 5p+0(1-p) = 5p and the expected gain from uncooperative action equals 10p+1(1-p) = 9p+1 [4]. We define as the precision parameter, which is inversely related to the variance of the error (2). For every λ, p = QRE(λ) could be found from the formula (1) from the solution of the Eq (2)

$$\frac{\exp\left(\lambda \times 5p\right)}{\exp\left(\lambda \times 5p\right)+\exp\left(\lambda \left(9p+1\right)\right)}=\frac{1}{1+\exp\left(\lambda \left(4p+1\right)\right)}$$ (2)

At λ = 0 the probability of the cooperative move by QRE(λ) equals 0.5 (the chaotic behavior). With an increase of λthe probability of cooperation by QRE(λ) decreases and within the limit λ→∞ strives to 0 and this corresponds to the single Nash equilibrium in PD. Thus, from the QRE position any percentage of cooperative moves less than 50% could be justified. It is quite suitable for games before Socialization. Mathematically that means the solution of the QRE(λ) = p equation is relative to the λ parameter for a given observed level of the cooperative moves p. In this case, this equation is easily solved:

$$\lambda =\frac{\ln(\frac{1-p}{p})}{4p+1}$$ (3)

We give all the solutions to this equation for the series of experiments (Table 2) in fall, 2015.

**Table 2:** The average level of cooperation and the QRE parameter for all the experiments in PD before the Socialization stage.

Experiment date	The average level of cooperation before Socialization (%)		Lambda, λ
	M	SD	
15.09.2015	0.121	0.13	1.334
21.09.2015	0.144	0.15	1.131
28.09.2015	0.409	0.31	0.139
05.10.2015	0.227	0.14	0.641
09.10.2015	0.364	0.21	0.228
12.10.2015	0.417	0.26	0.126
19.10.2015	0.356	0.26	0.244
26.10.2015	0.212	0.22	0.710

The table represents the average level of cooperation before Socialization and calculated Lambda for all experiments.

**Fig 3:**
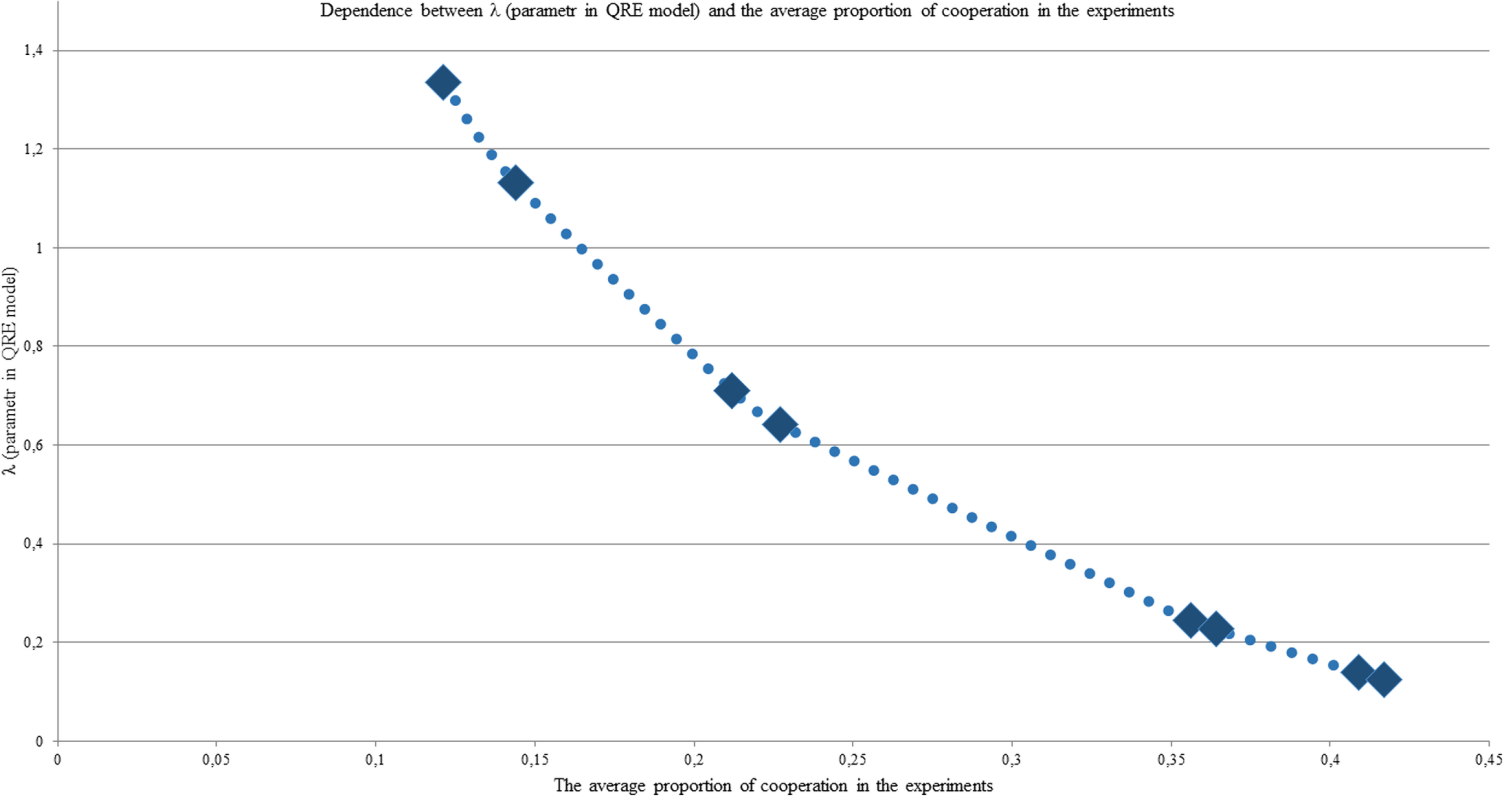
The average level of cooperation and the QRE parameter for all experiments in PD before the Socialization stage. The λparameter (y-axis) as a function of the average level of cooperation in every experiments (x-axis).

For clarity, we represent this dependence graphically (Fig 3).

We see that the maximum degree of cooperation before Socialization was achieved 12.10.2015 and is 41.7%, which corresponds to the significantly positive value λ = 0.126. The minimum degree of cooperation before Socialization was reached 15.09.2015 and is 12.1% at λ = 1.334 (Fig 3), which is far from the limit value. The average value of cooperation in the experiment series is 28% at λ = 0.44.

Thus, the calculations show that the behavior of the participants of the experiments in PD before Socialization is completely described by the QRE concept, which is the adopted deviation from the Nash equilibrium towards the easing of the requirements of the best answer.

However, after Socialization the situation radically changes.

As Table 3 shows, the level of cooperation after Socialization is over 50% in all the experiments, which is why the QRE concept is not fully applicable in this case.

**Table 3:** The average levels of the cooperative moves in PD after Socialization.

Experiment date	The average level of cooperation after Socialization (%)	
	M	SD
15.09.2015	0.741	0.35
21.09.2015	0.537	0.33
28.09.2015	0.639	0.43
05.10.2015	0.574	0.35
09.10.2015	0.528	0.29
12.10.2015	0.718	0.33
19.10.2015	0.796	0.27
26.10.2015	0.556	0.37

The table represents the average level of cooperation after Socialization.

This means that it is necessary to search for an alternative theoretical game model for the behavior of participants.

### QRE in the TG

Let us find the QRE for the TG, which was also in a series of experiments in fall, 2015.

Unlike the static PD game, TG is a dynamic game with perfect information. The QRE concept is theoretically applicable in this case too. Let k = 0,…,10 be the trust level of player 1, and n = 0,…,3k be the gratefulness level of player 2. For a given level of k,n the winning of player 1 is 10-n+k, and the winning of player 2 is 3k-n. According to QRE, the probability p_2_(k,n)of thanks of a level n for a given level of trust k is determined by the formula

$$p_{2}(k,n)=\frac{e^{\lambda (3k-n)}}{\sum_{i=0}^{3k}e^{\lambda (3k-i)}}$$ (4)

For this reason, the expected winning u_1_(k)of player 1 with a level of trust k is

$$u_{1}(k)=10-k+\sum_{n=0}^{3k}np_{2}(k,n)$$ (5)

Then the probability p_1_(k)of trust of level k in QRE is determined by the formula

$$p_{1}(k)=\frac{e^{\lambda u_{1}(k)}}{\sum_{i=0}^{10}e^{\lambda u_{1}(i)}}$$ (6)

To find the parameter λ according to the results of the experiment let us calculate the average levels of trust k* and thanks n* and compare them with the theoretical expected level of trust k(λ) and gratefulness n(λ), which are calculated as

$$k(\lambda )=\sum_{k=0}^{10}p_{1}(k)\cdot k,\;n(\lambda )=\sum_{k=0}^{10}\sum_{n=0}^{3k}p_{1}(k)\cdot p_{2}(k,n)\cdot n$$ (7)

Let us select parameter λ so that several levels (k(λ),n(λ)) would become as close as possible to the levels (k*,n*) observed in the experiment.

As the following results of calculations show, parameter λ even for games before Socialization is rather close to 0; thus the behavior of the participants according to QRE is treated as nearly chaotic for some experiments.

Table 4 shows the average values of trust and gratefulness for each experiment in the fall, 2015 series with the calculated parameter λ and approximate values of trust and gratefulness, which approximate the specified average values in the best way. The average of value λ for this series is estimated at 0.17, which is significantly lower than the average value λ obtained previously for the PD game in the same series of experiments. Hence, we can conclude that the QRE concept poorly explains the results of experiments even before Socialization.

**Table 4:** Average trust and gratefulness in comparison with QRE for the TG.

Experiment date	Average trust		Average gratefulness		Lambda	Forecast of trust	Forecast of gratefulness
	M	SD	M	SD			
15.09.2015	5.38	3.48	3.17	2.54	0.15	4.39	3.63
21.09.2015	4.25	2.68	1.98	2.15	0.23	3.67	2.43
05.10.2015	3.33	1.36	0.94	0.69	0.34	2.81	1.55
09.10.2015	5.58	2.74	4.12	3.27	0.11	4.70	4.40
12.10.2015	5.80	3.03	4.87	3.75	0.09	4.89	5.08
19.10.2015	5.81	3.32	6.88	3.29	0.03	5.08	6.86
26.10.2015	4.06	3.28	2.62	2.07	0.21	3.89	2.74

The table shows the average trust, average gratefulness, calculated Lambda, forecast of trust and forecast of gratefulness for all experiments.

### Model of iterated PD in Markov strategies

Let us construct and analyze a model of the iterated PD in Markov strategies. At the beginning, take into consideration the effect of the iterated PD several times with random partners. For simplicity let us assume that every participant responds only to the move made by his or her partner in the previous round. Such strategies are called Markov strategies or strategies with memory length equal to one [37,38]. Previously, the Markov chains were used more than once to find the equilibria for the Prisoner's Dilemma [19,37–40]. In [19] only equilibria for "good", cooperative strategies were found. Based on the past results, we also decided to apply Markov strategies for the theoretical justification of the experimental data. However, we were interested not in extreme cases, which lead to total cooperation rarely observed in experiments, but in the internal equilibrium points, in which both cooperation and betrayal are selected with positive probabilities.

For the PD game after Socialization, the following approach described in previous works [38,41–45] was suitable the most:

Let γ_i_ denote reciprocal cooperation, i.e. the probability that a participant i will act cooperatively after the previous round in which his or her partner played cooperatively.

Let α_i_ denote tolerance to defection, i.e. the probability that a participant i will act cooperatively after the previous round in which his or her partner played non-cooperatively.

For the given parameters of cooperation and tolerance of the pair of participants, we obtain a Markov process with a finite number of states [46–48]. In the stationary distribution, each player of the pair of participants will be in one of two possible states: {Cooperation, Defection}. The stationary probability p_i_^c^ for a participant i to be in a cooperative state depends on the stationary probability p_j_^c^ of a participant j≠i and strategic parameters of reciprocal cooperation γ_i_ and tolerance to defection α_i_ of a participant i in the following way

$$p_{i}^{c}=\gamma_{i}p_{j}^{c}+\alpha_{i}(1-p_{j}^{c}),\;i,j=1,2,\;j\neq i$$ (8)

For the given strategies {α_1_, α_2_, γ_1,_ γ_2_} of two unknowns participants (1 –the first participant and 2 –the second) this system of two linear equations with is easily solved in explicit form:

$$p_{i}^{c}=\frac{\alpha_{i}-\alpha_{j}(\alpha_{i}-\gamma_{i})}{1-(\alpha_{1}-\gamma_{1})(\alpha_{2}-\gamma_{2})},\;i=1,2$$ (9)

The composition of corresponding probabilities gives the stationary distribution for all four pairs of actions of the participants, and based on this distribution we can calculate the profits of the participants. Omitting the intermediate calculations, let us write the expression for the winning of participant 1:

$$U_{1}(p_{1}^{c},p_{2}^{c})=-4p_{1}^{c}p_{2}^{c}-p_{1}^{c}+9p_{2}^{c}+1$$ (10)

It should be remembered that (p_1_^c^, p_2_^c^) in their turn depend on {α_1_,α_2_,γ_1_,γ_2_} as indicated above. Therefore, there turns out to be some kind of a game in a normal form with nonlinear payoff functions. But a symmetric totally mixed Nash equilibria (in Markov strategies) {α, α, γ, γ} can be found in explicit form in this game:

$$5\alpha^{2}+9\gamma^{2}-14\alpha \gamma +14\alpha -10\gamma +1=0$$ (11)

**Fig 4:**
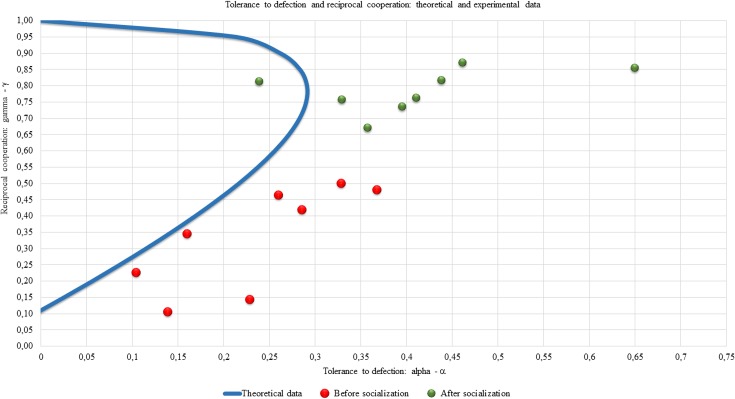
A set of symmetric equilibria of the Markov game on the plane tolerance-betrayal, reciprocal cooperation (theoretical result). Reciprocal cooperation γ (y-axis) as a function of the tolerance to the defection α(x-axis) for every experiment.

It can be checked whether this curve of the second order is a hyperbolic curve. Let us represent it in the intersection with a single square of tolerance and cooperation (Fig 4).

The upper point (0, 1) in Fig 4 corresponds to the standard tit-for-tat strategy with 100% reciprocal cooperation and zero tolerance to defection [49]. It is not an interior point of the space of strategic parameters, so the stationary distribution for the pairs of such strategies is determined uniquely and depends on the initial conditions. Without going into detail, let us assume that the participants always make a move cooperatively in the first round, then the pair of tit-for-tat strategies leads to complete cooperation.

For us, the equilibria with maximum tolerance to defection (0.3, 0.8) are of particular importance. We will treat a section of the hyperbolic curve below this point as equilibria before Socialization, and above the point as equilibria after Socialization. Let us pay attention to the fact that high levels of reciprocal cooperation (over 80%) are realized only during rather low tolerance.

## Results and discussion

Let us apply the theoretical calculations obtained to the experimental data. We will consider the data on the PD before and after Socialization.

Table 5 displays the cases of cooperative and non-cooperative moves, reciprocal cooperation and tolerance to defection in PD before and after Socialization. There Ncoop is the number of a partner's cooperative moves in the previous round; Nrecoop the number of cooperative moves in response to the cooperation in the previous round; Ndefault the number of a partner's non-cooperative moves in the previous round; and Ntolerant the number of cooperative moves after the partner's defection in the previous round.

**Table 5:** Number of cooperative and non-cooperative moves, reciprocal cooperation and tolerance to defection in PD before and after Socialization.

Experiment date	Before Socialization				After Socialization			
	Ncoop	Nrecoop	Ndefault	Ntolerant	Ncoop	Nrecoop	Ndefault	Ntolerant
15.09.2015	31	7	221	23	70	61	26	12
21.09.2015	19	2	101	14	53	39	43	17
28.09.2015	50	25	70	23	131	100	73	30
05.10.2015	28	4	92	21	119	90	85	28
09.10.2015	43	18	77	22	109	73	95	34
12.10.2015	52	25	68	25	147	120	57	25
19.10.2015	43	20	77	20	76	65	20	13
26.10.2015	26	9	94	15	112	91	92	22

The table contains the number of a partner's cooperative moves in the previous round (Ncoop); the number of cooperative moves in response to the cooperation in the previous round (Nrecoop); the number of a partner's non-cooperative moves in the previous round (Ndefault); and the number of cooperative moves after the partner's defection in the previous round (Ntolerant) for all experiments before and after Socialization.

This data allow us to assess the parameters α and γ for each experiment before and after Socialization. It is natural to assess value α as $\frac{\mathrm{Ntolerant}}{\mathrm{Ndefault}}$, and value γ as $\frac{\mathrm{Nrecoop}}{\mathrm{Ncoop}}$. This assessment is presented in Table 6.

**Table 6:** Assessment of the parameters of reciprocal Socialization and tolerance to defection according to the results of the experiments before and after Socialization.

Experiment date	Before Socialization		After Socialization	
	Alpha, α	Gamma, γ	Alpha, α	Gamma, γ
15.09.2015	0.104	0.226	0.462	0.871
21.09.2015	0.139	0.105	0.395	0.736
28.09.2015	0.329	0.500	0.411	0.763
05.10.2015	0.228	0.143	0.329	0.756
09.10.2015	0.286	0.419	0.358	0.670
12.10.2015	0.368	0.481	0.439	0.816
19.10.2015	0.260	0.465	0.650	0.855
26.10.2015	0.160	0.346	0.239	0.813

The table contains tolerance to defection (Alpha) and reciprocal cooperation (Gamma) for all experiments before and after Socialization.

**Fig 5:**
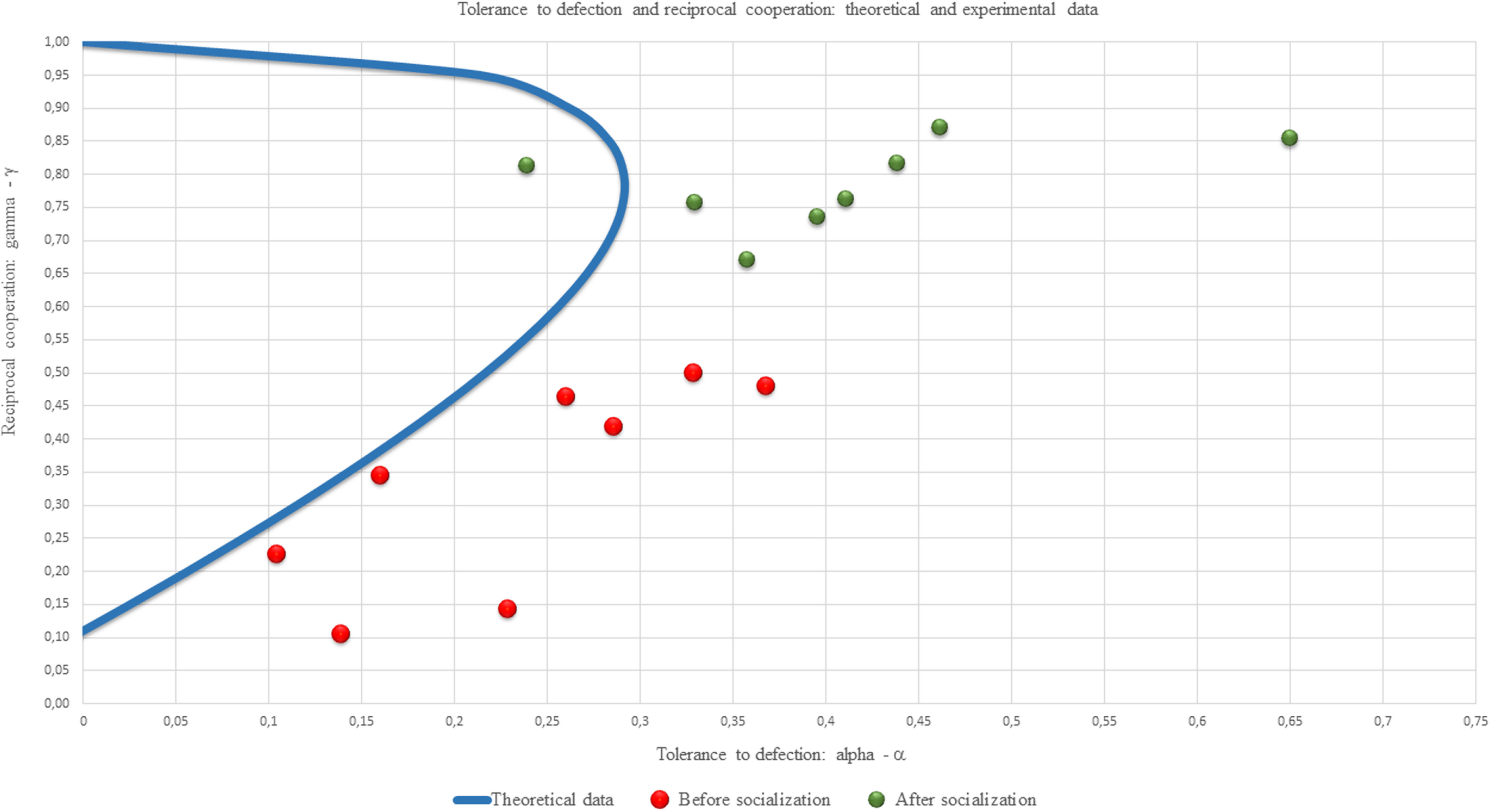
Tolerance to defection and reciprocal cooperation: Theory and experiment. Reciprocal cooperation γ (y-axis) as a function of the tolerance of the defection α (x-axis) for every experiments: comparison of the theoretical and experimental data.

Now let us placed the obtained pairs of assessment from Table 6 on the plane (α,γ) together with a section of the hyperbolic curve falling within the unit square (Fig 5).

From the data shown in Fig 5 we can formulate the following results:

Result 1. An increase in the responding cooperation level after Socialization.

The separation of the points in Fig 5 into two vertical clusters is evident.

Result 2. Observable tolerance to betrayal exceeds theoretical tolerance in the equilibrium in nearly all the experiments (15 points out of 16).

All the points except one in Fig 5 lie to the right of the hyperbolic curve.

Result 3. More than a third of the experiments (6 points out of 16) are consistent with the theory.

Result 4. Almost all the experimental data are in a position to the ε-equilibria for the iterated PD in Markov strategies.

Let us set off the horizontal distance (tolerance) from the experimental points to the hyperbolic curve to prove results 3 and 4.

The experiments in which the distance to the theoretical equilibria of tolerance is less than 0.1 are highlighted in Table 7.

**Table 7:** The distance of tolerance to defection from the theoretical equilibria to the experimental data before and after Socialization.

Experiment date	Before Socialization	After Socialization
15.09.2015	***0*.*03269024***	0.185075
21.09.2015	0.14237892	0.106521
28.09.2015	0.11205629	0.119693
05.10.2015	0.20802336	***0*.*038593***
09.10.2015	0.10754084	***0*.*081434***
12.10.2015	0.1597411	0.148679
19.10.2015	***0*.*05905651***	0.367935
26.10.2015	***0*.*01923364***	***-0*.*0512***

The table displays the distance of tolerance to defection from the theoretical equilibria to the experimental data before and after Socialization.

Now let us check how significant the deviation in profit is from the theoretical equilibria. To do this, we will make the following calculation.

1. Let us calculate the point on the hyperbolic curve with the same reciprocal cooperation for each experimental point.
2. For each point on the hyperbolic curve let us calculate the values of equilibrium of the player's profit.
3. Let us treat each experimental point as a deviation of one of the players on tolerance to betrayal from the equilibrium, considering that the other player adheres to the equilibrium. Let us calculate the decrease in the profit of the deviated player in the percentage of his equilibrium profit.

The results of this calculation are presented in Table 8.

**Table 8:** The deviation of the winning from the theoretical equilibria to the experimental data before and after Socialization.

Experiment date	Before Socialization	After Socialization
15.09.2015	0.03%	0.31%
21.09.2015	0.92%	0.16%
28.09.2015	0.25%	0.19%
05.10.2015	1.68%	0.02%
09.10.2015	0.26%	0.11%
12.10.2015	0.51%	0.25%
19.10.2015	0.08%	1.01%
26.10.2015	0.01%	0.04%

The table shows the deviation of the winning from the theoretical equilibria to the experimental data before and after Socialization.

**Fig 6:**
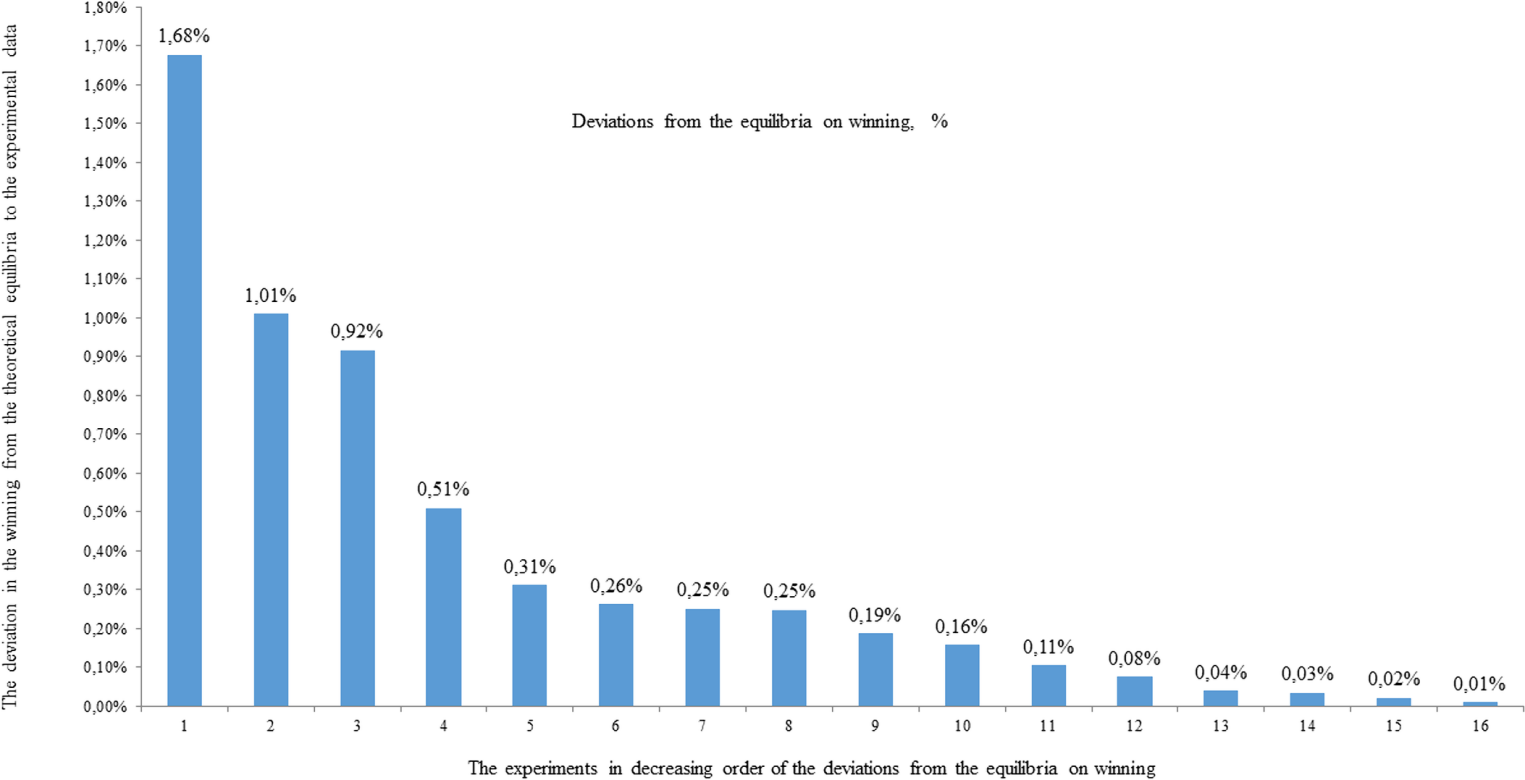
Deviation of experiments from the equilibria on winning in percentages. The deviation in winning in the experimental data from the theoretical equilibrium (y-axis) during the experiments in decreasing order of the deviations from the equilibria in winning (x-axis).

For clarity, let us order the values obtained in Table 8 in descending order and present them graphically (Fig 6).

Fig 6 shows that the maximum deviation from the equilibria on winning is only 1.7%, and this deviation is no more than 0.5% in 75% of cases. This means that nearly all the experiments are in a position of ε-equilibria for the iterated PD in Markov strategies. This result is fundamental.

## Conclusions

This research applied a QRE model to the results of an experiment designed to study cooperation and trust under the influence of social interaction. The resulting data were divided into two categories: before and after social interaction. The peculiarity of the results is that the data on cooperation after Socialization in the Socialization stage are significantly different from those of the experiments in PD. The calculations showed that the behavior of the participants before Socialization could be described with the QRE concept, which is an accepted deviation from the concept of Nash equilibrium that weakens the requirements of the best response. However, the standard QRE approach cannot be applied to describing the behavior of the participants after Socialization. Therefore, we have proposed a variant of the description of equilibria in the iterated PD in Markov strategies. For this game repeated in Markov strategies, we managed to explicitly find all the equilibria with a positive probability of reciprocal cooperation and tolerance to betrayal. The primary result is that all the experiments are in a position of ε-equilibria for the repeated PD game in Markov strategies. There remain questions of theoretical justification of the results of such games as the Trust Game and Ultimatum Game, the experimental data of which do not correspond to known theoretical game models in the framework of our research on the influence of social interaction.

## References

[1] Lukinova E, Babkina T, Myagkov M. Choosing Your Teammates Creates Social Identity and Keeps Cooperation Rates High. World Academy of Science, Engineering and Technology International Journal of Economics and Management Engineering. 2015; 7 Available from: goo.gl/dWxk9L

[2] Roth AE. Individual rationality and Nash’s solution to the bargaining problem. Math Oper Res. 1977;2: 64–65.

[3] Bernheim BD, Peleg B, Whinston MD. Coalition-Proof Nash Equilibria I. Concepts. J Econ Theory. 1987;42: 1–12. doi: 10.1016/0022-0531(87)90099-8

[4] McKelvey RD, Palfrey TR, Weber RA. The effects of payoff magnitude and heterogeneity on behavior in 2×2 games with unique mixed strategy equilibria. J Econ Behav Organ. 2000;42: 523–548. doi: 10.1016/S0167-2681(00)00102-5

[5] Myerson RB. Refinements of the Nash equilibrium concept. Int J Game Theory. 1978;7: 73–80. doi: 10.1007/BF01753236

[6] Dong Y, Li C, Tao Y, Zhang B. Evolution of Conformity in Social Dilemmas. PLoS ONE. 2015;10: e0137435 doi: 10.1371/journal.pone.0137435 26327137 10.1371/journal.pone.0137435 PMC4556697

[7] Goeree JK, Holt CA. Asymmetric inequality aversion and noisy behavior in alternating-offer bargaining games. Eur Econ Rev. 2000;44: 1079–1089. doi: 10.1016/S0014-2921(99)00048-3

[8] Tumennasan N. To err is human: Implementation in quantal response equilibria. Games Econ Behav. 2013;77: 138–152. doi: 10.1016/j.geb.2012.10.004

[9] Campbell R, Sowden L. Paradoxes of rationality and cooperation: Prisoner’s dilemma and Newcomb’s problem. UBC Press; 1985.

[10] Colman AM. Cooperation, psychological game theory, and limitations of rationality in social interaction. Behav Brain Sci. 2003;26: 139–153. doi: 10.1017/S0140525X03000050 14621510 10.1017/s0140525x03000050

[11] Boyd R, Lorberbaum JP. No pure strategy is evolutionarily stable in the repeated Prisoner’s Dilemma game. Nature. 1987;327: 58–59. doi: 10.1038/327058a0

[12] Englmaier F, Wambach A. Optimal incentive contracts under inequity aversion. Games Econ Behav. 2010;69: 312–328. doi: 10.1016/j.geb.2009.12.007

[13] Ostrom E, Walker J. Trust and reciprocity: Interdisciplinary lessons for experimental research. Russell Sage Foundation; 2003.

[14] Starr JA, MacMillan IC. Resource Cooptation Via Social Contracting: Resource Acquisition Strategies for New Ventures. Strateg Manag J. 1990;11: 79–92.

[15] McWilliams A, Siegel DS, Wright PM. Corporate Social Responsibility: Strategic Implications*. J Manag Stud. 2006;43: 1–18. doi: 10.1111/j.1467-6486.2006.00580.x

[16] Roberts RW. Determinants of corporate social responsibility disclosure: An application of stakeholder theory. Account Organ Soc. 1992;17: 595–612. doi: 10.1016/0361-3682(92)90015-K

[17] Choi S, Gale D, Kariv S. Social learning in networks: a Quantal Response Equilibrium analysis of experimental data. Rev Econ Des. 2012;16: 135–157. doi: 10.1007/s10058-012-0122-x

[18] Goeree JK, Holt CA, Palfrey TR. Quantal Response Equilibrium and Overbidding in Private-Value Auctions. J Econ Theory. 2002;104: 247–272. doi: 10.1006/jeth.2001.2914

[19] Akin E. Good strategies for the iterated prisoner’s dilemma. ArXiv Prepr ArXiv12110969 V2. 2013;

[20] Nowak M, Sigmund K. A strategy of win-stay, lose-shift that outperforms tit-for-tat in the Prisoner’s Dilemma game. Nature. 1993;364: 56–58. doi: 10.1038/364056a0 8316296 10.1038/364056a0

[21] Cesarini D, Dawes CT, Fowler JH, Johannesson M, Lichtenstein P, Wallace B. Heritability of cooperative behavior in the trust game. Proc Natl Acad Sci. 2008;105: 3721–3726. doi: 10.1073/pnas.0710069105 18316737 10.1073/pnas.0710069105 PMC2268795

[22] Delgado MR, Frank RH, Phelps EA. Perceptions of moral character modulate the neural systems of reward during the trust game. Nat Neurosci. 2005;8: 1611–1618. doi: 10.1038/nn1575 16222226 10.1038/nn1575

[23] Fischbacher U. z-Tree: Zurich toolbox for ready-made economic experiments. Exp Econ. 2007;10: 171–178.

[24] Xia C-Y, Meng X-K, Wang Z. Heterogeneous coupling between interdependent lattices promotes the cooperation in the prisoner’s dilemma game. PloS One. 2015;10: e0129542 doi: 10.1371/journal.pone.0129542 26102082 10.1371/journal.pone.0129542 PMC4477883

[25] Ma Z-Q, Xia C-Y, Sun S-W, Wang L, Wang H-B, Wang J. Heterogeneous link weight promotes the cooperation in spatial prisoner’s dilemma. Int J Mod Phys C. 2011;22: 1257–1268.

[26] Wang J, Xia C, Wang Y, Ding S, Sun J. Spatial prisoner’s dilemma games with increasing size of the interaction neighborhood on regular lattices. Chin Sci Bull. 2012;57: 724–728.

[27] Meng X-K, Xia C-Y, Gao Z-K, Wang L, Sun S-W. Spatial prisoner’s dilemma games with increasing neighborhood size and individual diversity on two interdependent lattices. Phys Lett A. 2015;379: 767–773.

[28] Babkina T, Myagkov M, Lukinova E, Peshkovskaya A, Menshikova O, Berkman ET. Choice of the group increases intra-cooperation. CEUR-Workshop. 2016;1627: 13–24. Available from: https://cla2016.hse.ru/data/2016/07/24/1119025624/EEML2016.pdf.

[29] Lukinova E, Myagkov M. Impact of Short Social Training on Prosocial Behaviors: An fMRI Study. Front Syst Neurosci. 2016;10. 10.3389/fnsys.2016.00060 PMC4932112 27458349

[30] Peshkovskaya AG, Babkina TS, Myagkov MG, Kulikov IA, Ekshova KV, Harriff K. The socialization effect on decision making in the Prisoner’s Dilemma game: An eye-tracking study. PloS One. 2017;12: e0175492 doi: 10.1371/journal.pone.0175492 28394939 10.1371/journal.pone.0175492 PMC5386283

[31] Berkman ET, Lukinova E, Menshikov I, Myagkov M. Sociality as a Natural Mechanism of Public Goods Provision. PLoS ONE. 2015;10: e0119685 doi: 10.1371/journal.pone.0119685 25790099 10.1371/journal.pone.0119685 PMC4366235

[32] Mckelvey RD, Palfrey TR. Quantal response equilibria for extensive form games. Exp Econ. 1998;1: 9–41. doi: 10.1007/BF01426213

[33] McKelvey RD, Palfrey TR. Quantal response equilibria for normal form games. 1993;

[34] Goeree JK, Holt CA, Palfrey TR. Regular Quantal Response Equilibrium. Exp Econ. 2005;8: 347–367. doi: 10.1007/s10683-005-5374-7

[35] Zhuang Q, Di Z, Wu J. Stability of Mixed-Strategy-Based Iterative Logit Quantal Response Dynamics in Game Theory. PLoS ONE. 2014;9: e105391 doi: 10.1371/journal.pone.0105391 25157502 10.1371/journal.pone.0105391 PMC4144851

[36] Zhang B. Social Learning in the Ultimatum Game. PLoS ONE. 2013;8: e74540 doi: 10.1371/journal.pone.0074540 24023950 10.1371/journal.pone.0074540 PMC3762740

[37] Kandori M, Mailath GJ, Rob R. Learning, Mutation, and Long Run Equilibria in Games. Econometrica. 1993;61: 29 doi: 10.2307/2951777

[38] Press WH, Dyson FJ. Iterated Prisoner’s Dilemma contains strategies that dominate any evolutionary opponent. Proc Natl Acad Sci. 2012;109: 10409–10413. doi: 10.1073/pnas.1206569109 22615375 10.1073/pnas.1206569109 PMC3387070

[39] Karandikar R, Mookherjee D, Ray D, Vega-Redondo F. Evolving aspirations and cooperation. J Econ Theory. 1998;80: 292–331.

[40] Selten R, Stoecker R. End behavior in sequences of finite Prisoner’s Dilemma supergames A learning theory approach. J Econ Behav Organ. 1986;7: 47–70.

[41] Hauert C, Schuster HG. Effects of increasing the number of players and memory size in the iterated Prisoner’s Dilemma: a numerical approach. Proc R Soc Lond B Biol Sci. 1997;264: 513–519.

[42] Doebeli M, Hauert C. Models of cooperation based on the Prisoner’s Dilemma and the Snowdrift game. Ecol Lett. 2005;8: 748–766.

[43] Ebel H, Bornholdt S. Coevolutionary games on networks. Phys Rev E. 2002;66: 056118. 10.1103/PhysRevE.66.056118 12513567

[44] Hauert C, Michor F, Nowak MA, Doebeli M. Synergy and discounting of cooperation in social dilemmas. J Theor Biol. 2006;239: 195–202. doi: 10.1016/j.jtbi.2005.08.040 16242728 10.1016/j.jtbi.2005.08.040 PMC2891160

[45] Milinski M, Wedekind C. Working memory constrains human cooperation in the Prisoner’s Dilemma. Proc Natl Acad Sci. 1998;95: 13755–13758. 9811873 10.1073/pnas.95.23.13755 PMC24892

[46] Baum LE, Petrie T. Statistical Inference for Probabilistic Functions of Finite State Markov Chains. Ann Math Stat. 1966;37: 1554–1563.

[47] Tauchen G. Finite state markov-chain approximations to univariate and vector autoregressions. Econ Lett. 1986;20: 177–181. doi: 10.1016/0165-1765(86)90168-0

[48] Wheeler R, Narendra K. Decentralized learning in finite Markov chains. IEEE Trans Autom Control. 1986;31: 519–526.

[49] Axelrod R. The evolution of strategies in the iterated prisoner’s dilemma. Dyn Norms. 1987; 1–16.

